# Macrocytic Anaemia: Not Always a Straightforward Diagnosis

**DOI:** 10.7759/cureus.23152

**Published:** 2022-03-14

**Authors:** Andreia M Teixeira, Bernardo Macedo, Carla P Fontes, Micaela Manuel

**Affiliations:** 1 Internal Medicine, Centro Hospitalar de Entre Douro e Vouga, Santa Maria da Feira, PRT

**Keywords:** polyglandular syndrome, pernicious anaemia, microangiopathy, megaloblastic anaemia, macrocytic anaemia

## Abstract

Macrocytosis is defined as a mean corpuscular volume greater than 100 femtolitres (fL). There are several causes for macrocytic anaemia, and they can be divided into megaloblastic or non-megaloblastic anaemia. Vitamin B12 deficiency is one of the most common causes of megaloblastic anaemia.

The cause of vitamin B12 deficiency must be evaluated including the presence of pernicious anaemia as it could alter the treatment and follow-up.

Pernicious anaemia can be associated with other autoimmune diseases constituting polyglandular syndromes.

## Introduction

Macrocytosis is defined as a mean corpuscular volume greater than 100 femtolitres (fL) occurring in about 3% to 4% of the general population [1,2].

There are several causes for macrocytosis and generally, they are divided into megaloblastic and non-megaloblastic anaemia. This division depends on the peripheral blood smear findings as megaloblastic processes are characterized by macro-ovalocytes and hypersegmented neutrophils which, theoretically, are absent in non-megaloblastic processes [3,4]. 

Megaloblastic processes are generally due to vitamin B12 and folate deficiencies. Vitamin B12 deficiency is common in the general population and can affect about 15% of the elderly [5].

Moreover, haemolysis is another cause of non-megaloblastic macrocytosis [1]. Reticulocytosis, increased lactate dehydrogenase, unconjugated bilirubin and decreased haptoglobin levels are diagnostic predictors of haemolysis [6]. The direct antiglobulin test differentiates from immune and non-immune causes for haemolysis. 

Intramedullary haemolysis can occur in about 1.5% of patients with megaloblastic anaemia due to ineffective erythropoiesis. But in this situation, reticulocytosis, schistocytes and direct antiglobulin tests are not present [7].

Although a rare condition, pernicious anaemia should be considered before vitamin B12 deficiency [7]. There is an association of pernicious anaemia with other autoimmune disorders such as thyroiditis, Addison’s disease, vitiligo and type 1 diabetes. The existence of at least two autoimmune endocrinopathy makes the diagnosis for polyglandular autoimmune syndrome [8]. 

## Case presentation

A 75-year-old man with a history of arterial hypertension, dyslipidaemia and vitiligo presented to consultation with symptoms of asthenia, extremities paraesthesia and memory loss. Physical examination showed pale skin and mucosa, atrophic glossitis and skin discoloration suggestive of vitiligo without other findings, including altered blood pressure. He was medicated daily with a statin and a calcium channel blocker with no history of new drug therapy, dietary changes, or blood loss.

Initial blood tests showed macrocytic anaemia with a haemoglobin of 7.9 g/dL with a mean cell volume of 108 fL, white cell count of 4.8x10^9^/L and a platelet count of 11x10^9^/L. There was an elevation of total bilirubin (1.12 mg/dL), serum lactate dehydrogenase (1018 U/L), reticulocytosis (reticulocyte index of 2.39) and decreased haptoglobin with negative direct antiglobulin test. Homocysteine was elevated. Renal function was normal with a serum creatinine of 0.7 mg/dL. Blood smear exhibited macro-ovalocytes, hypersegmented neutrophils and a few schistocytes.

Vitamin B12 was decreased (serum concentration of 89 pg/mL) with folic acid at the normal range. Measurement of intrinsic factor and parietal cells antibodies were positive and gastric biopsy showed atrophic gastritis without metaplasia and negativity for *Helicobacter pylori*, performing the diagnosis of pernicious anaemia. Measurement of thyroid hormones revealed subclinical hypothyroidism with positive thyroid peroxidase antibodies with normal adrenocorticotropic hormone (ACTH) and morning serum cortisol levels and negative results for islet cells and glutamic acid decarboxylase (GAD) antibodies. 

Intramuscular therapy with vitamin B12 was initiated. A few weeks later the patient had recovered from all symptoms and blood tests showed normal haemoglobin, platelet and reticulocyte count, blood smear, bilirubin and haptoglobin measurement.

## Discussion

This paper reports a case of macrocytic anaemia due to vitamin B12 deficiency.

Our patient presented with macrocytic anaemia, thrombocytopenia and signs of haemolysis that could lead to the diagnosis of thrombotic thrombocytopenic purpura, a life-threatening condition in need of plasma exchange. In cases of vitamin B12 deficiency, intramedullary haemolysis can occur causing “pseudo-thrombotic microangiopathy”[5]. This emphasizes the importance of conjugating all data including clinical symptoms as the patient presented with paraesthesia of the extremities and memory loss which occurs frequently in pernicious anaemia [2,9]. In this case, a blood smear revealed only a few schistocytes with a great number of macro-ovalocytes and hypersegmented neutrophils that is typical of megaloblastic anaemia, a normal renal function, and a low measurement of vitamin B12 which reinforced the diagnosis of megaloblastic anaemia.

There are several causes of vitamin B12 deficiency (Table 1) and although pernicious anaemia is a rare disorder it is seen more commonly in adults over 70 years old [9]. Thus, its evaluation must be pursued. In most cases, pernicious anaemia is an autoimmune disease characterized by chronic atrophic gastritis and impaired absorption of vitamin B12 from the terminal ileum due to the presence of antibodies against intrinsic factors [1]. It can be caused by non-autoimmune gastritis secondary to *H. pylori* infections [1].

**Table 1:** Differential diagnosis of macrocytosis

Differential diagnosis of macrocytosis	
Megaloblastic (vitamin B12 and acid folic deficiencies)	Atrophic gastritis
	Enteral malabsorption
	Human immunodeficiency virus treatment
	Anticonvulsants
	Primary bone marrow disorders
	Nitrous oxide abuse
	Inherited disorders
	Atrophic gastritis
	Enteral malabsorption
	Human immunodeficiency virus treatment
Non-megaloblastic	Alcohol abuse
	Medication side effects (cholestyramine, metformin, methotrexate, trimethoprim/sulfamethoxazole)
	Myelodysplasia
	Hypothyroidism
	Liver disease
	Haemolysis
	Haemorrhage
	Chronic obstructive pulmonary disease
	Splenectomy
False elevations	Cold agglutinins
	Hyperglycaemia
	Marked leucocytosis
	Cold agglutinins

There is no gold standard test for pernicious anaemia. Although antibodies to intrinsic factors are highly specific (95% to 100%), a negative test does not exclude the diagnosis due to its low sensitivity. Anti-gastric parietal cell antibodies have variable specificity (50% to 100%) [9]. Our patient had intrinsic factor and parietal cell antibodies with atrophic gastritis in the gastric biopsy confirming the diagnosis of pernicious anaemia. Patients with chronic atrophic autoimmune gastritis have an increased risk of gastric adenocarcinomas and gastric carcinoid tumours with the need for close follow-ups to detect precancerous changes [2,9].

There is an association among autoimmune disorders such as autoimmune thyroiditis, Addison’s disease, vitiligo, type 1 diabetes and pernicious anaemia [9]. Our patient fulfilled the criteria for autoimmune polyglandular syndrome type 3 (Table 2) since autoimmune thyroiditis, pernicious anaemia and vitiligo with normal adrenal function were present [8]. 

**Table 2:** Types and common phenotypes of autoimmune polyglandular syndrome Table 2 presents all four types of autoimmune polyglandular syndrome listing more common phenotypes in each type. Type 3 can be divided into four subcategories depending on the combination of autoimmune conditions with autoimmune thyroiditis always being present  [10,11].

Types and common phenotypes of autoimmune polyglandular syndrome	
Type 1	Candidiasis, Addison disease, hypoparathyroidism
Type 2	Addison disease, autoimmune thyroid disease and/or type 1A diabetes mellitus in combination with other conditions (Addison disease must be present)
Type 3	Autoimmune thyroid disease and other autoimmune conditions (Addison disease is not present)
Type 4	Two or more autoimmune conditions not classified elsewhere

## Conclusions

This case highlights the importance of conjugating clinical, physical and laboratory data that could lead to a straightforward diagnosis as vitamin B12 deficiency can mimic microangiopathies that could lead to unnecessary treatments. It also reinforces the need to pursue the investigation of coexisting autoimmune endocrinopathies when presented with an autoimmune condition, even in asymptomatic patients, as the treatment and follow-up may take a different course.
